# Single *GNAS* Droplet-Based Digital Polymerase Chain Reaction Analysis of Pancreatic Cyst Fluid: An Effective Up-Front Strategy for Mucinous Cyst Diagnosis by Endoscopic Ultrasound-Guided Fine-Needle Aspiration

**DOI:** 10.14309/ctg.0000000000000887

**Published:** 2025-07-07

**Authors:** Isis K. Araujo, Guillem Soy, Angels Ginès, Oriol Sendino, Glòria Fernández-Esparrach, Cristina Sánchez-Montes, Miriam Cuatrecasas, Ivan Archilla, Carla Montironi, Alós Silvia, Fabio Ausania, Manuel Domínguez-Fraile, Verónica Villagrasa, Mónica López-Guerra, Dolors Colomer, Eva C. Vaquero

**Affiliations:** 1 Gastroenterology Department, ICMDM, Hospital Clínic, Barcelona, Spain;; 2 University of Barcelona (UB), Barcelona, Spain;; 3 Institut d’Investigacions Biomèdiques August Pi i Sunyer (IDIBAPS), Barcelona, Spain;; 4 Centro de Investigación Biomédica en Red en Enfermedades Hepáticas y Digestivas (CIBEREHD), Madrid, Spain;; 5 Pathology Department, Biomedical Diagnostic Center Hospital Clinic, Barcelona, Spain;; 6 Department of Hepatic, Pancreatic, Biliary and Transplant Surgery, Hospital Clínic de Barcelona, Barcelona, Spain;; 7 Hematopathology Unit, Pathology Department, Biomedical Diagnostic Center, Hospital Clinic, Barcelona, Spain; 8 Centro de Investigación Biomédica en Red en Cáncer (CIBERONC), Madrid, Spain.

**Keywords:** pancreatic cystic lesions, molecular analysis, *GNAS*, droplet-based digital PCR, ddPCR

## Abstract

**INTRODUCTION:**

Accurate diagnosis of mucinous pancreatic cystic neoplasms (mPCNs) remains a clinical challenge. This study investigated the utility of single *GNAS* droplet-based digital polymerase chain reaction (ddPCR) analysis as a novel approach to refine the diagnostic accuracy of mPCNs using endoscopic ultrasound-guided fine-needle aspiration (EUS-FNA).

**METHODS:**

Patients who underwent EUS-FNA and *GNAS* pancreatic cyst fluid (PCF) analyses for pancreatic cystic lesion (PCL) assessment were prospectively enrolled. Cysts were categorized as mPCNs, non-mPCNs, or inconclusive PCLs (iPCLs) by integrating increasing information levels: high-resolution imaging and non-DNA PCF features (level 1), *GNAS* PCF analysis (level 2), and surgical pathology (level 3).

**RESULTS:**

One hundred forty patients were included, 25 of whom underwent pancreatic surgery. Level 1 identified 68 mPCNs (49%), 24 non-mPCNs (17%), and 48 iPCLs (34%). *GNAS* mutations were detected in 42 of 68 (62%) mPCNs, 1 of 24 (4%) non-mPCNs, and 16 of 48 (33%) iPCLs. Level 2 increased mPCN detection to 62% and reduced iPCLs by one-third. Mutated *GNAS* showed 66% sensitivity for diagnosing mPCNs in the whole cohort and 65% in resected cases, outperforming both imaging and non-DNA PCF mucinous criteria, with 100% specificity and limited concordance with carcinoembryonic antigen, cytology, and fluid viscosity, highlighting its complementary diagnostic value. Cost-effectiveness simulations for iPCLs demonstrated that *GNAS*-ddPCR significantly reduced diagnostic costs by 24% compared with next-generation sequencing testing.

**DISCUSSION:**

Single *GNAS-*ddPCR analysis in PCF supported mPCNs diagnosis in 62% of cases and uncovered 33% of iPCLs as mPCNs with 100% specificity. It adds complementary value to standard cyst fluid markers offering a simple and cost-effective tool for improving PCL diagnosis by EUS-FNA.

## Graphical abstract

**Figure:**
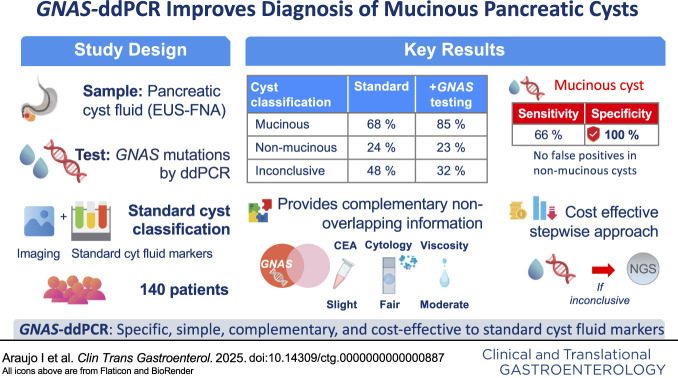

## INTRODUCTION

Pancreatic cystic lesions (PCLs) are increasingly common in adults and often discovered incidentally during abdominal imaging (1,2). Accurate cyst categorization is essential for effective decision making. Excluding clinically insignificant incidental cysts (3,4), mucinous pancreatic cystic lesions (mPCNs) are the most common type of cysts (2). Notably, mPCNs account for over 60% of PCLs examined using endoscopic ultrasound (EUS)-based techniques (5). Because mPCNs require clinical monitoring or surgical removal because of their premalignant nature, their recognition is crucial. Most mPCNs are intraductal papillary mucinous neoplasms (IPMN), which constitute approximately 80% of incidental PCLs (6) and 89% (7)—96% (8) of cysts under surveillance. The remaining mPCNs are mucinous cystic neoplasms (MCNs), which account for 2% of all PCLs (6,7). Non-mucinous cysts (non-mPCNs) include a variety of neoplastic and non-neoplastic entities with different behaviors.

Given the challenges in PCL categorization, with some being misclassified (9,10) or remaining inconclusive (11) despite high-resolution imaging and standard pancreatic cystic fluid (PCF) analysis, there is a clear need for improved diagnostic methods. DNA-based targeted sequencing of driver gene mutations in PCF samples has become a valuable tool for evaluating PCLs, surpassing the standard diagnostic methods for cyst categorization and advanced neoplasia detection (12–19). DNA-targeted sequencing panels typically include genes for PCL categorization (e.g., *GNAS*, *KRAS*, *BRAF*, *HRAS*, *RNF43*, *VHL*, *MEN1*, and *CTNNB1*) and high-risk prediction (e.g., *TP53*, *PIK3CA*, *PTEN*, *AKT1*, *CDKN2A*, and *SMAD4*). However, a major challenge in the widespread adoption of PCF DNA testing is establishing a cost-effective approach. It is worth considering that if the purpose of EUS-guided fine-needle aspiration (EUS-FNA) is to determine the PCL type, diagnostic genes would be sufficient. Given the high prevalence of IPMN among PCLs, a reasonable diagnostic approach would involve initially searching for mucinous DNA biomarkers using a dedicated method, such as *GNAS* analysis, before escalating to NGS for inconclusive or equivocal cysts, or when assessing malignancy risk.

Somatic activating point mutations in codon 201 (*R201C* and *R201H*) of the alpha subunit of the stimulatory GTP-binding protein (*GNAS*) are critical mucinous biomarkers because of their absolute specificity for mucinous cysts (20). Rare mutations, such as *R201S*, *R844H*, and *Q227L*, have also been identified in a low proportion of IPMN (11,21). Studies on surgical pathology cohorts have reported *GNAS* mutations in PCF samples in 45–66% of IPMN cases (13,17,18,20,22–25), occasionally in MCN (23–25), and absent in non-mPCNs. The absolute specificity of *GNAS* mutations for mucinous cysts not only underscores its diagnostic accuracy but also highlights its potential to significantly improve the diagnostic workup of PCLs.

The detection of genomic alterations in PCF requires high-throughput techniques to address low-allele frequency variants with limited DNA quantities. Droplet-based digital polymerase chain reaction (ddPCR) is a cost-effective ultrasensitive method that is useful for detecting hotspot gene mutations with a low detection limit and provides rapid results within hours. This technique is suitable for analyzing *GNAS* and *KRAS* missense mutations in PCF and secretin-stimulated pancreatic fluid samples (26–28). However, the diagnostic efficacy of *GNAS*-ddPCR in routine PCF samples remains to be determined.

To address these diagnostic challenges, we designed our study to critically evaluate the effectiveness of single *GNAS* ddPCR analysis on PCF as a diagnostic tool for mucinous cysts.

## METHODS

### Study design

This is a retrospective analysis of a prospectively collected single-center cohort conducted at Hospital Clínic, with Ethical Committee approval (IRB 2019-1-119). Written informed consent was obtained from all participants. From November 2015 to September 2022, patients with PCLs who underwent EUS-FNA and PCF-*GNAS* testing were consecutively enrolled. Experienced endoscopists performed EUS-FNA following established protocols (29). Clinicopathological data, high-resolution imaging (EUS, magnetic resonance imaging, computed tomography), and PCF findings (viscous consistency, carcinoembryonic antigen [CEA] concentration, cytopathology) were reviewed by expert gastroenterologists using electronic medical records. PCF samples were analyzed for DNA in all cases and for CEA and cytomorphology when sufficient material was available.

### Cyst categorization

**Figure 1.**
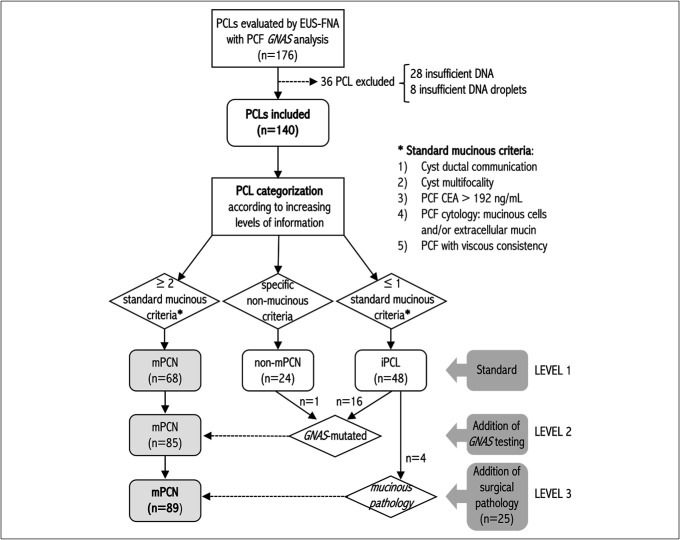
Flowchart of the study cohort, illustrating PCL categorization across levels 1, 2, and 3 based on the diagnostic criteria applied. PCL, pancreatic cystic lesion.

Cysts were classified as mPCN, non-mPCN, or inconclusive (iPCL) using a stepwise approach (Figure 1). Level 1 relied on imaging and standard PCF data, requiring mPCNs to meet at least 2 mucinous criteria (cyst multifocality, ductal communication, viscous PCF, CEA concentration >192 ng/mL, or mucinous cytology) to minimize misclassification. Non-mPCNs were categorized based on characteristic imaging features and clinical context. Cysts were categorized as iPCLs when they exhibited one or no mucinous features and lacked definitive characteristics to confidently classify them as either mucinous or nonmucinous. Level 2 incorporated *GNAS* results, and level 3 included surgical pathology diagnoses.

### Cytomorphological analysis

Fresh PCF samples were subjected to cytospin slide preparation for Papanicolaou staining and/or cell block formalin fixation using hematoxylin and eosin staining. Pathology reports followed the WHO Reporting System for Pancreaticobiliary Cytopathology (29), classifying cases as follows: I (inadequate), II (benign), III (atypical), IV (PanLow neoplasm), V (PanHigh neoplasm, including high-grade dysplasia and invasive adenocarcinoma in mucinous cysts), VI (suspicious for malignancy), and VII (malignant, including nonmucinous pancreatic ductal adenocarcinoma). PanLow and PanHigh classifications were based on thick, colloid-like mucin and/or mucinous neoplastic epithelium with varying degrees of atypia.

### CEA concentration analysis

CEA was measured in fresh PCF with a Cobas analyzer using an electrochemiluminescence immunoassay based on the sandwich principle.

### DNA analysis

DNA was extracted from fresh or frozen PCF samples using a Qiagen DNA Easy Blood Kit. For formalin-fixed surgical pancreatic tissues, the GeneRead DNA FFPE kit (Qiagen, Hilden, Germany) was used, with uracil DNA glycosylase pretreatment to reduce cross-linking false positives before PCR. DNA concentration was measured using a NanoDrop 2000 and Qubit dsDNA high-sensitivity assay kit (Thermo Fisher Scientific, Waltham, MA).

### Droplet-based digital PCR

*GNAS* mutations *R201H* and *R201C* were analyzed using a QX200 Droplet Digital PCR system and validated using a ddPCR mutation assay (Bio-Rad, Hercules, CA). The reaction mixture was prepared according to the manufacturer's instructions. Positive events for FAM or HEX channels and the fractional abundance of mutations were calculated using the QuantaSoft (ver. 1.7) software based on Poisson distribution. The target copies per droplet were adjusted using software to fit the Poisson model with 95% confidence. Fractional abundance was calculated as [A/(A + B)], where A is a mutant and B is the copy number of wild-type allele. The detection limit was defined as the lowest number of droplets with mutant alleles above the blank limit, with at least 3 positive droplets for the GNAS mutation and a minimum of 500 droplets. All reagents and software were from Bio-Rad Laboratories (Hercules, CA).

### Statistical analysis

Quantitative variables were presented as mean ± SD or median and interquartile range, whereas categorical variables as counts and percentages. The Mann-Whitney *U* test was used to compare continuous variables, and the Fisher exact test was used to assess the relationships between categorical variables. Spearman rank correlation coefficient (Spearman ρ) was used for ordinal variables. Diagnostic performance was assessed using cutoff values from receiver operating characteristic curve analysis, defined by the highest sensitivity and specificity near the upper-left corner. Sensitivity, specificity, predictive values, and accuracy were calculated. Statistical significance was set at *P* < 0.05. Concordance between categorical diagnostic tests was evaluated using Cohen Kappa coefficient. Analyses were conducted using Stata V.17 statistical software (StataCorp, College Station, TX) and GraphPad Prism 9.5.1 for MacOS (GraphPad Software, Boston, MA).

## RESULTS

### Patient characteristics and study cohort description

A total of 176 patients with PCLs who underwent EUS-FNA and PCF DNA testing were initially enrolled (Figure 1). Thirty-six patients were excluded because of insufficient DNA (28 patients) or inadequate droplet numbers (8 patients) for ddPCR analysis. The final cohort comprised 140 patients (69 men, 49%; 71 women, 51%) aged 62 ± 13 years. The DNA concentration in PCF samples ranged from 0.02 to 219 ng/μL, with a median of 4.86 ng/μL.

### Level 1: cyst categorization based on imaging and standard ancillary PCF information

High-resolution imaging and standard PCF information (gross consistency, CEA concentration, and cytopathology) identified 49% (68/140) of cases as mPCNs and 17% (24/140) as non-mPCNs. The remaining 34% (48/140) were classified as iPCLs, with 26 (54%) not meeting the standard mucinous criteria and 22 (46%) meeting only one. Table 1 presents the data for the entire cohort, with each cyst category classified as diagnostic level 1.

**Table 1.** Patient characteristics and cyst features of 140 cases categorized as mPCN, non-mPCN, and iPCL at level 1

Characteristics	All cysts (n = 140, 100%)	mPCN (n = 68, 49%)	Non-mPCN (n = 24, 17%)	iPCL (n = 48, 34%)
Age at EUS-FNA, yr (mean ± SD)	61.4 (13)	65 (11)	55.4 (13.7)	59.3 (13.6)
Gender, male/female, n (%)	69/71 (49/51)	38/30 (56/44)	12/12 (50/50)	19/29 (40/60)
Incidental, n (%)	101 (72)	53 (78)	9 (38)	39 (81)
Symptomatic presentation, n (%)	39 (28)	15 (22)	15 (62)	9 (19)
Cyst type, n (%)				
BD-IPMN		51 (75)	—	—
Mixed-IPMN		14 (21)	—	—
MCN		3 (4)	—	—
Pseudocysts		—	11 (46)	—
SCN		—	5 (21)	—
Groove pancreatitis-associated cysts		—	2 (8)	—
Chronic pancreatitis-associated cysts		—	1 (4)	—
PDAC with cystic component		—	3 (13)	—
SPN		—	1 (4)	—
pNET		—	1 (4)	—
Inconclusives		—	—	48 (100)
Cyst size on EUS, mm, median (IQR)	26.8 (17–32)	23.5 (14–30)	36.3 (19–50)	27.0 (18–31)
Cyst septations on EUS, n (%)	64/137 (47)	29/66 (44)	13/24 (54)	22/47 (47)
Cyst location, n (%)				
Head or uncinate	70 (50)	39 (57)	13 (54)	18 (38)
Neck	9 (6)	6 (9)	0	3 (6)
Body	36 (26)	18 (27)	5 (21)	13 (27)
Tail	25 (18)	5 (7)	6 (25)	14 (29)
Cyst multifocality, n (%)				
Yes	61 (44)	47 (69)	4 (17)	10 (21)
No	79 (56)	21 (31)	20 (83)	38 (79)
Cyst ductal communication, n (%)				
Yes	59 (42)	52 (77)	3 (12)	4 (8)
No	81 (58)	16 (23)	21 (88)	44 (92)
PCF consistency, n (%)				
Available, n	131	63	20	47
Viscous	41 (31)	35 (56)	0	7 (15)
Nonviscous	90 (69)	28 (44)	20 (100)	40 (85)
PCF CEA, n (%)				
Available, n	94	39	16	39
Mucinous (>192 ng/mL)	26 (28)	24 (62)	1 (6)	1 (3)
Nonmucinous (<192 ng/mL)	68 (72)	15 (38)	15 (94)	38 (97)
Concentration (ng/mL), median (IQR)	39 (2.4–267)	369 (103–1,697)	15 (2.1–75)	3.7 (0.9–36.8)
PCF cytopathology, n (%)				
Available, n	113	57	20	36
Mucinous (WHO IV and V)	29 (26)	28 (49)	1 (5)	0
Nonmucinous (WHO I, II, III, VI)	84 (74)	29 (51)	19 (95)	36 (100)
PCF WHO cytopathological category, n (%)				
Available, n	113	57	20	36
I (nondiagnostic)	30 (27)	13 (19)	4 (20)	13 (36)
II Negative for malignancy	48 (42)	15 (22)	10 (50)	23 (64)
III (Atypical)	1 (0.8)	1 (1)	0	0
IV (PanLow)	22 (19)	22 (32)	0	0
V (PanHigh)	5 (4)	4	1 (5)^a^	0
VI (positive for malignancy)	7 (6)	2	5 (25)	0
Surgical resection, n (%)	25 (18)	12 (18)	1 (4)	12 (25)
*GNAS*, n (%)				
Mutated	59 (42)	42 (62)	1 (4)	16 (33)
Wild-type	81 (58)	26 (38)	23 (96)	32 (67)

The WHO numbers (I-VI) displayed on the PCF cytopathology variable indicate the diagnostic category in accordance with the revised WHO System for Reporting Pancreatobiliary Cytopathology (29).

mPCN, mucinous pancreatic cystic neoplasm; EUS-FNA, endoscopic ultrasound-guided fine-needle aspiration; PCF, pancreatic cyst fluid; CEA, carcinoembryonic antigen; IPMN, intraductal papillary mucinous neoplasm; MCN, mucinous cystic neoplasm; PDAC, pancreatic ductal adenocarcinoma; pNET, pancreatic neuroendocrine tumor; SCN, serous cystic neoplasm; SPN, solid pseudopapillary neoplasm.

a Patient with chronic pancreatitis with false cytological diagnosis of adenocarcinoma.

### Level 2: cyst categorization after incorporating *GNAS* PCF testing

**Figure 2.**
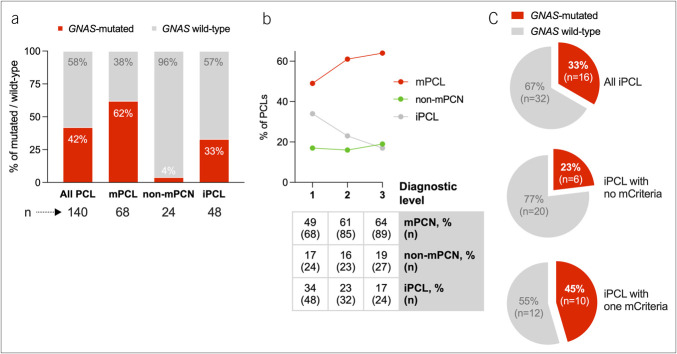
Prevalence of *GNAS* mutations in PCF obtained by EUS-FNA and cyst categorization according to diagnostic criteria. (**a**) Bar graph showing the proportion of *GNAS*-mutated PCF samples within the total study cohort and across the 3 PCL categories defined at diagnostic level 1. (**b**) Line graph illustrating changes in PCL classification across 3 diagnostic levels. Level 1 is based on imaging and standard PCF features. Level 2 incorporates *GNAS* mutations status, reclassifying *GNAS*-mutated non-mPCN and iPCL as mPCN. Level 3 includes histologic diagnoses from 25 surgically resected lesions. A summary table is included. (**c**) Pie charts showing the proportion of *GNAS*-mutated iPCL cases (from level 1), stratified by the presence of zero or one of mucinous criteria. EUS-FNA, endoscopic ultrasound-guided fine-needle aspiration; iPCL, inconclusive pancreatic cystic lesion; mPCN, mucinous pancreatic cystic neoplasm; PCF, pancreatic cyst fluid.

Of the 140 PCF samples, 59 (42%) had *GNAS* mutations and 81 (58%) were wild-type. *GNAS* mutations were found in 62% (42/68) of mPCNs, 4% (1/24) of non-mPCNs, and 33% (16/48) of iPCLs (Figure 2a). The non-mPCN case with mutant *GNAS* was suspected to be a serous cystic neoplasm (SCN) based on a honeycomb-like pattern on EUS.

Given that mutated *GNAS* is a categorical mucinous feature, 16 of 48 iPCLs (33%) and 1 of 24 non-mPCNs with mutant *GNAS* (4%) were reclassified as mucinous, improving mPCNs detection from 49% to 61%. Figure 2b shows the cyst reclassification using incremental levels of information.

We analyzed *GNAS* mutations in iPCLs, dichotomized by the presence or absence of standard mucinous criteria (Figure 2c, Supplementary Table S1, http://links.lww.com/CTG/B338). *GNAS* mutations were found in 23% (6/26) of the cases lacking mucinous criteria and in 45% (10/22) of the cases meeting one mucinous criterion. Among the latter, *GNAS* supported mucinous diagnosis in 50% (2/4) of cysts with ductal communication, 30% (3/10) of multifocal cysts, and 71% (5/7) of viscous PCF. Notably, 79% (38/48) of iPCL had non-mucinous CEA concentrations (<192 ng/mL), with 34% (13/38) showing *GNAS* mutations (Supplementary Table S1, http://links.lww.com/CTG/B338).

In summary, targeted *GNAS* analysis supported a mucinous diagnosis in 62% of the lesions, changed one nonmucinous cyst diagnosis, and clarified the diagnosis in one-third of the inconclusive cysts. These findings underscore the enhanced diagnostic precision obtained by incorporating *GNAS* mutation analysis.

### *GNAS* analysis of surgically resected cases

**Figure 3.**
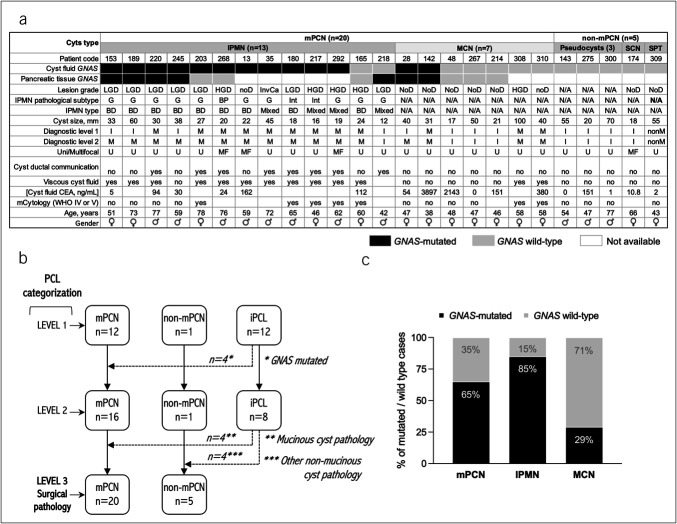
(**a**) Overview of the clinicopathological features and *GNAS* mutation status in 25 surgically resected cases. (**b**) Flowchart of the subcohort of surgically resected cases, showing PCL categorization based on the diagnostic criteria. (**c**) Frequency of *GNAS* mutations in presurgical PCF samples from 20 mPCNs with confirmed surgical pathology. mPCN, mucinous pancreatic cystic neoplasm; PCF, pancreatic cyst fluid; PCL, pancreatic cystic lesion.

Surgical pathology data were available for 25 of 140 patients (18%), with a median interval of 3.4 months (interquartile range 1.9–7.55) between *GNAS* testing and surgery. Figure 3a summarizes the resected cases, including *GNAS* mutations detected in preoperative PCF DNA and in paired resected pancreatic tissue, along with the clinicopathological data.

Of the 25 resected cases, 20 (80%) were mPCNs (13 IPMNs, 7 MCNs) and 5 were non-mucinous lesions (3 pseudocysts, 1 SCN, and 1 solid pseudopapillary neoplasm) (Supplementary Table S2, http://links.lww.com/CTG/B338). Histology matched the presurgical diagnoses in 17 cases (68%), whereas 8 cases (32%) were iPCLs with wild-type *GNAS* before resection (Figure 3b). The pathological diagnoses of these iPCL included 1 IPMN, 3 MCNs, 3 pseudocysts, and 1 SCN.

Preoperative PCF samples showed *GNAS* mutations in 13 of 20 mPCN cases (65%): 11 of 13 IPMNs (85%) and 2 of 7 MCNs (29%) (Figure 3c). No *GNAS* mutations were found in non-mPCN PCF samples. Molecular analysis of resected tissues from 8 IPMN and 5 MCN cases showed *GNAS* genotype concordance between PCF and tissue in 10 of 13 cases (77%). Three cases were discordant (Figure 3a): 2 had *GNAS* mutations in PCF but not in tissue (cases 203 and 268), and one had *GNAS* mutations in tissue but not in PCF (case 218).

In summary, the detection of *GNAS* mutations in PCF samples was significantly correlated with the presence of mPCNs, underscoring the utility of *GNAS*-ddPCR in enhancing diagnostic accuracy.

### Level 3: cyst cohort categories after incorporating pathology data

Histopathologic analysis of 25 resected cases led to the reclassification of 8 iPCLs with wild-type *GNAS*: 4 as mPCNs (1 IPMN, 3 MCNs) and 4 as non-mPCNs (1 SCN, 3 pseudocysts). The final cohort comprised 89 mPCNs (64%; 82 IPMNs, 7 MCNs), 27 non-mPCNs (19%), and 24 iPCLs (17%) (Table 2). Supplementary Table S3 (http://links.lww.com/CTG/B338) details the clinical and cystic characteristics of IPMNs identified at level 3.

**Table 2.** PCL categorization at level 3 and frequency of *GNAS* mutations in each category

Types of PCLs diagnosed at level 3	All, n (%)	*GNAS-*mutated, n (%)
mPCN	89 (64)	59/89 (66)
IPMN	82 (59)	57/82 (70)
MCN	7 (5)	2/7 (29)
Non-mPCN	27 (19)	0
Pseudocysts	14 (10)	0
SCN	5 (3.6)	0
PDAC cystic component	3 (2.1)	0
Groove pancreatitis-associated cysts	2 (1.4)	0
Chronic pancreatitis-associated cyst	1 (0.7)	0
pNET	1 (0.7)	0
SPN	1 (0.7)	0
iPCL	24 (17)	0

iPCL, inconclusive pancreatic cystic lesion; IPMN, intraductal papillary mucinous neoplasm; MCN, mucinous cystic neoplasm; mPCN, mucinous pancreatic cystic neoplasm; PDAC, pancreatic ductal adenocarcinoma; pNET, pancreatic neuroendocrine tumor; SCN, serous cystic neoplasm; SPN, solid pseudopapillary neoplasm.

To clarify the diagnostic relevance of our classification, particularly in non-mPCNs, we stratified these lesions by clinical behavior (Supplementary Table S4, http://links.lww.com/CTG/B338). Non-mPCN were subclassified as benign/non-neoplastic (e.g., SCNs, pseudocysts) or nonmucinous neoplastic (e.g., pancreatic ductal adenocarcinoma, solid pseudopapillary neoplasm, and pancreatic neuroendocrine tumor). Notably, no *GNAS* mutations were detected in either subgroup, reinforcing its high specificity for mucinous cysts regardless of malignant potential or management.

### *GNAS* mutation status in mPCNs at level 3

Among the 89 mPCN cases at level 3, 59 (66%) had *GNAS* mutations in the PCF. In the IPMN subgroup, 57 of 82 cases (70%) harbored mutations (Supplementary Figure S1A, http://links.lww.com/CTG/B338): *R201C* in 22 cases (39%), *R201H* in 21 (37%), and both mutations in 14 (25%) (Supplementary Figure S1B, http://links.lww.com/CTG/B338). Mutant allele frequencies (MAF) ranged from 0.02% to 49%, with a median of 1.15%. No significant difference in MAF was found between *R201C* and *R201H* (median 1.11% vs 1.64%, *P* = 0.377) (Supplementary Figure S1C, http://links.lww.com/CTG/B338). Most cases (73%) had an MAF < 5% (Supplementary Figure S1D, http://links.lww.com/CTG/B338).

In MCNs, *GNAS* mutations were detected in the PCF of 2 of the 7 lesions (Figure 3a). Case 28 had both *R201C* (0.9% MAF) and *R201H* (0.5% MAF), whereas case 142 had *R201H* (0.2% MAF). PCF mutations were confirmed in the resected tissue, although only *R201C* was detected in case 28.

### Association of *GNAS* mutation status with clinical and cyst features in IPMN cases

The *GNAS* genotype in IPMN cases showed no significant association with age, sex, abdominal symptoms, and history of pancreatitis, diabetes, extrapancreatic malignancies, or cyst characteristics (Supplementary Table S3, http://links.lww.com/CTG/B338). However, *GNAS*-mutated cases had a higher frequency of viscous consistency (59% vs 27%, *P* = 0.011) and a lower frequency of mucinous CEA (31% vs 79%, *P* = 0.003) than wild-type cases. CEA concentration was also significantly lower in *GNAS*-mutated cases (median 94 vs 1,441 ng/mL, *P* < 0.002) (Supplementary Figure S3, http://links.lww.com/CTG/B338).

A higher but nonsignificant trend of malignancy was observed in wild-type IPMNs than that in *GNAS*-mutated cases (16% vs 9%, *P* = 0.335) (Supplementary Table S3, http://links.lww.com/CTG/B338).

No associations were found between *GNAS* MAF values and patient or cyst characteristics in *GNAS*-mutated IPMN cases (Supplementary Table S5, http://links.lww.com/CTG/B338).

Owing to the limited number of MCN cases, statistical associations for *GNAS* were not analyzed.

### Diagnostic performance of PCF *GNAS* testing and other mucinous criteria in IPMN cases

**Figure 4.**
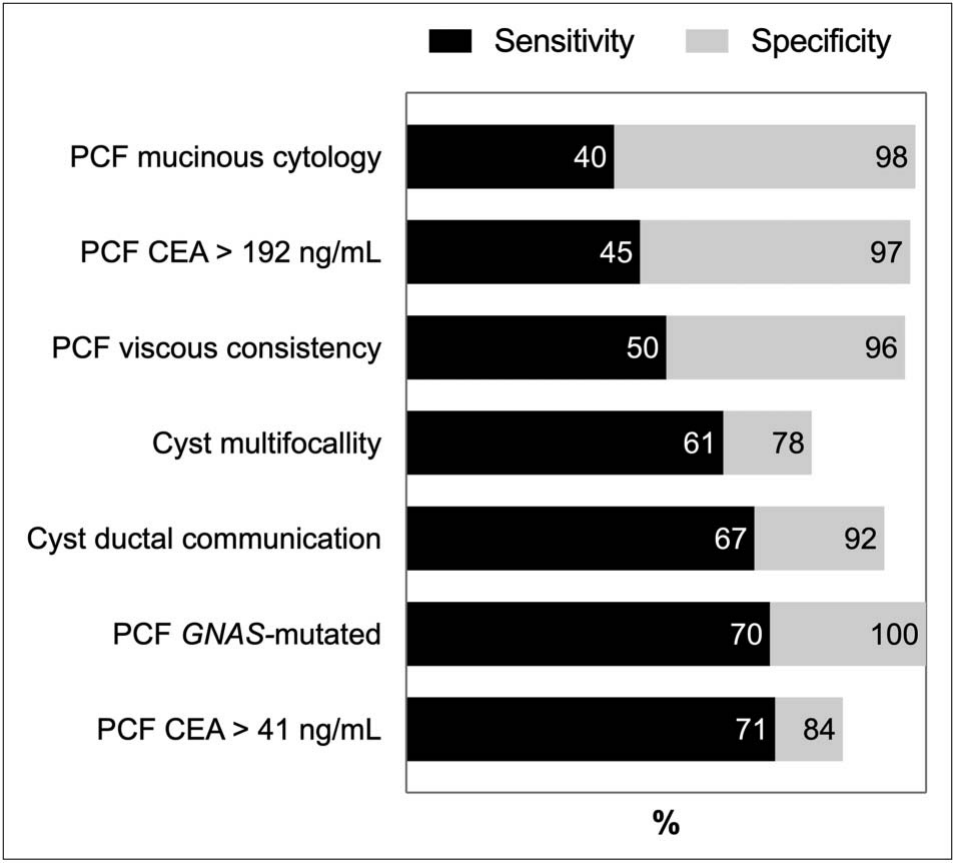
Sensitivity and specificity of mucinous imaging features and ancillary PCF criteria for distinguishing IPMNs from other pancreatic cystic lesions. Values were calculated for the entire study cohort (n = 140) and categorized according to diagnostic level 3. PCF, pancreatic cyst fluid; IPMN, intraductal papillary mucinous neoplasm.

In the 82-IPMN subcohort, *GNAS* mutations yielded the highest sensitivity (70%) and perfect specificity (100%) among mucinous criteria (Figure 4, Supplementary Table S6, http://links.lww.com/CTG/B338). By contrast, cytopathology, CEA >192 ng/mL, and viscous consistency were highly specific (98%, 97%, 96%, respectively) but had lower sensitivity (40%, 45%, 50%, respectively). Receiver operating characteristic analysis identified an optimal CEA cutoff of 41 ng/mL, with 71% sensitivity, similar to *GNAS*, but a reduced specificity of 84%.

In surgical cases (Supplementary Table S7, http://links.lww.com/CTG/B338), preoperative *GNAS* mutations and viscous consistency had the highest sensitivity for mPCNs (65% and 68%, respectively), compared with mucinous cytology (47%) and CEA >192 ng/mL (20%), all with 100% specificity. Lowering the CEA cutoff to >41 ng/mL increased sensitivity (67%) but decreased specificity (75%). Overall, among surgically confirmed PCLs, all PCF mucinous criteria were 100% specific for mPCNs, with *GNAS* mutations and viscous consistency offering the highest sensitivity. A summary of *GNAS* sensitivity across all diagnostic subgroups is provided in Supplementary Table S8 (http://links.lww.com/CTG/B338).

### Concordance between *GNAS* mutations and standard fluid-based markers

**Figure 5.**
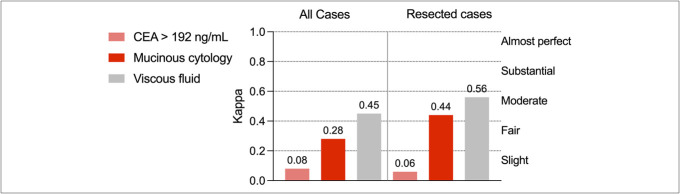
Concordance (Cohen Kappa) between GNAS mutations and standard fluid-based markers—CEA >192 ng/mL, mucinous cytology, and viscous cyst fluid—in all cases and in surgically resected patients. Interpretation thresholds for Kappa values are indicated as reference. See Supplementary Tables S8–S13 (http://links.lww.com/CTG/B338) for data. CEA, carcinoembryonic antigen.

We assessed the concordance between *GNAS* mutations and 3 standard mucinous fluid-based markers. Concordance with mucinous cytology was 66.4% (Cohen Kappa = 0.28), indicating fair agreement; with CEA >192 ng/mL, 58.5% (Kappa = 0.08), indicating slight agreement; and with fluid consistency, 73.8% (Kappa = 0.45), indicating moderate agreement (Figure 5, Supplementary Tables S9–S14, http://links.lww.com/CTG/B338).

Similar results were observed in the subset of patients who underwent surgical resection, with concordance values suggesting fair-to-moderate agreement across all 3 markers.

These findings highlight that *GNAS* provides complementary, nonoverlapping diagnostic information, which may be particularly useful in cases with inconclusive or discordant fluid-based results.

### Exploratory analysis of long-term outcomes according to *GNAS* mutation status in mPCNs

An exploratory analysis of 89 patients with mPCNs was performed to assess the incidence of advanced neoplasia arising during follow-up after the initial EUS-FNA, according to *GNAS* mutation status. Although limited by small numbers, neoplastic progression during follow-up occurred in 1 of 47 *GNAS*-mutated cases (2.1%) and in 2 of 21 *GNAS* wild-type cases (9.5%) (*P* = 0.22). Detailed data are presented in Supplementary Table S15 (http://links.lww.com/CTG/B338).

### Cost-effectiveness simulation of a stepwise molecular approach in inconclusive PCLs

We evaluated the financial implications of *GNAS*-ddPCR as an initial diagnostic step for iPCLs by comparing universal next-generation sequencing (NGS) with a stepwise strategy. Because the primary purpose of DNA analysis in this study was to categorize cysts, only iPCLs require extended NGS testing, making them the focus of this cost-effectiveness simulation.

A *GNAS*-ddPCR test costs €65 per patient, whereas a 12-gene NGS panel, currently in development at our institution, costs approximately €700. Of the 48 study cases (34%) categorized as iPCLs, 32 (67%) were *GNAS* wild type and would subsequently require NGS testing. Universal NGS for all 48 iPCLs would cost €33,600. By contrast, the stepwise approach involved *GNAS*-ddPCR for all 48 cases (€3,120), with NGS for the 32 wild-type cases (€22,400), totaling €25,520 and saving €8,080 (24% reduction) compared with universal NGS.

These findings highlight the cost-efficiency of using *GNAS*-ddPCR as an initial molecular test in the diagnostic workflow for iPCLs, optimizing resource allocation while maintaining accuracy.

## DISCUSSION

In this study, we demonstrate that the detection of *GNAS* mutations in PCF by ddPCR is a highly specific and clinically valuable tool for the characterization of PCLs. Our findings show that *GNAS* mutation analysis offers diagnostic information that is complementary—rather than redundant—to traditional mucinous features such as cytology, CEA levels, and fluid viscosity, as reflected by the limited concordance observed between these modalities. These results support the incorporation of *GNAS* testing into the diagnostic algorithm for PCLs, particularly when conventional fluid markers are inconclusive or discordant.

Our results align with those of prior studies demonstrating the utility of *GNAS* testing for mucinous cyst diagnosis and suggest a potentially more cost-effective, targeted approach. Previous research has primarily focused in surgical cohorts, introducing selection bias, because most PCL do not undergo surgical resection. Few studies have evaluated *GNAS* testing in nonsurgical clinical practice, and all have used multigene panels (11,15–17,19). By contrast, our study supports a stepwise diagnostic strategy, beginning with targeted *GNAS* testing and progressing to broader sequencing when necessary. In our series, *GNAS*-ddPCR resolved the diagnosis in 16 of 48 inconclusive cases, avoiding the need for DNA sequencing to categorize the lesion. The remaining 32 inconclusive cases without *GNAS* mutations would have required sequencing of other genes, such as *VHL*, *MEN1*, and *CTNNB1*.

Although a detailed cost-analysis was beyond the scope of this study, preliminary estimates suggest that ddPCR could be significantly less expensive than sequencing, warranting further economic evaluation in future studies. These cost-savings underscore the financial viability of *GNAS*-ddPCR as an initial diagnostic step. As the cost-effectiveness simulation analysis performed in this study focused on inconclusive cysts, future prospective studies evaluating universal ddPCR followed by targeted NGS in negative cases could further inform optimal diagnostic strategies.

Another aim of this study was to assess the sensitivity and specificity of *GNAS* mutations in PCF samples using ddPCR. Previous research has indicated a pooled sensitivity for mutated *GNAS* in PCF of 46% (39–56%) in mucinous neoplasms and 56% (45–66%) in IPMN through DNA-targeted sequencing (15,16,18–23). Our results showed a slightly higher *GNAS* mutation rate with ddPCR, with 65% of mucinous cysts and 70% of IPMN testing positive. This may be attributed to the superior sensitivity of ddPCR for detecting low-frequency DNA mutations compared with NGS. ddPCR has a detection limit as low as 0.002–0.05% (30,31) whereas NGS typically has a limit of approximately 1% (31). In addition, ddPCR has been shown to detect mutations missed by NGS (27,32).

*GNAS* mutations are primarily associated with mPCNs, typically exclusive to IPMN and absent in MCN (13,15,17,18,20) However, our study identified 2 MCN cases with *GNAS* mutations in both PCF and resected specimens. Prior studies have also reported *GNAS* mutations in MCN (23–25) suggesting that *GNAS* mutations may serve as broader biomarkers for mucinous cystic lesions beyond IPMNs.

*GNAS* is often analyzed along *KRAS* (11,15–17,22,23) or other *MAPK* pathway genes (*BRAF, HRAS, NRAS*) (18,33) to improve the detection of IPMN and MCN. Although *KRAS* mutations are more prevalent than *GNAS* mutations in mucinous PCLs (47–86% vs 39–56%), *GNAS* is more specific. *KRAS* can also be found in pseudocysts and retention cysts (23), pancreatic neuroendocrine tumors (23), pancreatic ductal adenocarcinoma (13), and metastatic cystic neoplasms (18). *KRAS* is also suitable for ddPCR. Van Huijgevoort et al demonstrated that ddPCR analysis of *KRAS* in PCF samples has a sensitivity of 63%, outperforming NGS (22%) (24,25,27). Future studies evaluating *GNAS* and *KRAS* ddPCR testing in PCF will be valuable.

Another secondary objective was to evaluate *GNAS*'s diagnostic performance compared with other mucinous criteria. Our results indicate that *GNAS* mutations have higher sensitivity and specificity for IPMN than mucinous imaging features and standard PCF ancillary markers. Only CEA>41 ng/mL showed comparable sensitivity for IPMN (71%) and mPCN (73%) as *GNAS* (70% and 65%, respectively) but lower specificity than CEA>192 ng/mL. These findings support the use of cohort-specific CEA thresholds. Recent studies have reported wide variability in optimal CEA cutoffs, reinforcing its role as an adjunctive rather than definitive diagnostic tool (23,34,35). It is worth noting that not all mPCNs carry *GNAS* mutations, and this marker should therefore be used as part of a multimodal diagnostic strategy integrating imaging, cytology, and fluid markers. These findings are consistent with previous studies showing *GNAS*'s superior diagnostic performance over imaging, viscosity, CEA, and cytology (17,18,23). This is further supported by the limited concordance observed between *GNAS* mutations and traditional fluid-based markers (CEA, cytology, and viscosity), highlighting the complementary, nonoverlapping diagnostic value of *GNAS* analysis.

To further explore the diagnostic specificity of *GNAS* and address the heterogeneity of the non-mPCN group, we performed a supplementary analysis subclassifying lesions into benign/non-neoplastic and nonmucinous neoplasms with potential surgical indication. None of the lesions in either subgroup harbored *GNAS* mutations, further supporting its specificity as a mucinous marker.

Beyond its diagnostic role, *GNAS* has not been firmly linked to advanced neoplasia, and its long-term prognostic value remains unexplored. Although findings in resected IPMNs suggest more favorable histology in *GNAS*-mutated cases (36,37), our exploratory data show a trend toward lower malignant progression in surveillance. Further studies are warranted.

This study has some limitations. Only 18% of PCL cases had pathologically confirmed diagnoses, meaning that *GNAS* specificity was assumed to be 100% based on its established association with mucinous cysts. However, the extensive literature supports this specificity. Cyst fluid glucose was not routinely collected during the study period. Recent studies support its value as a diagnostic marker, and it may be considered in future research. In addition, the study was conducted in a single center, underscoring the need for external validation in different clinical settings where EUS assessment of PCLs may vary. Finally, although the cohort was prospectively collected, the retrospective nature of the analysis may be subject to limitations related to data completeness and heterogeneity in clinical documentation.

In conclusion, our study highlights the value of integrating a single *GNAS* analysis into routine EUS-FNA diagnostics for PCLs, aiding the validation or identification of mucinous cysts when imaging or PCF parameters are inconclusive. This molecular approach offers a practical initial strategy in clinical practice and may help limit cases requiring NGS analysis.

## CONFLICTS OF INTEREST

**Guarantor of the article:** Eva C. Vaquero, MD, PhD.

**Specific author contributions:** E.C.V., A.G., I.K.A., G.S.: concept and design of the research; E.C.V., A.G., I.K.A., G.S., O.S., G.F.-E., M.D.-F., D.C., M.L.-G., V.V., I.K.A., F.A., M.C., D.C., M.L.-G.: implementation of the research; I.K.A. and E.C.V.: analysis and interpretation of the data; E.C.V.: drafting the manuscript; E.C.V., I.K.A., G.S., A.G., O.S., G.F.-E., D.C., M.L.-G.: critical revision of the manuscript. All authors approved the final version of the manuscript.

**Financial support:** Supported by CIBEREHD institutional funding (not linked to a specific grant).

**Potential competing interests:** None to report.

**Study Highlights**

**WHAT IS KNOWN**

✓ *GNAS* mutations are specific biomarkers of mucinous pancreatic cysts.
✓ *GNAS* analysis of pancreatic cystic fluid using DNA sequencing is limited by its high cost.
✓ Droplet-based digital polymerase chain reaction (ddPCR) is a low-cost, highly sensitive method that has not yet been evaluated for routine *GNAS* analysis of pancreatic cystic fluid.

**WHAT IS NEW HERE**

✓ *GNAS*-ddPCR is a sensitive and feasible method for detecting mutations in cystic fluid.
✓ *GNAS*-ddPCR strengthens mucinous cyst diagnosis and identifies mucinous lesions among cysts with inconclusive fluid analysis
✓ This study supports a stepwise diagnostic approach, starting with targeted testing and escalating to sequencing, when necessary.

## Supplementary Material

## ABBREVIATIONS:

AUC: area under the curve
BD-IPMN: branch-duct intraductal papillary mucinous neoplasm
CEA: carcinoembryonic antigen
CT: computerized tomography
ddPCR: droplet-based digital polymerase chain reaction
EUS: endoscopic ultrasound
EUS-FNA: endoscopic ultrasound-guided fine-needle aspiration
IQR: interquartile range
iPCL: inconclusive pancreatic cystic lesion
IPMN: intraductal papillary mucinous neoplasm
MAF: mutant allele frequency
MCN: mucinous cystic neoplasm
NPV: negative predictive value
mPCN: mucinous pancreatic cystic neoplasm
MRI: magnetic resonance imaging
non-mPC: nonmucinous pancreatic cystic lesion
PanLow: pancreatobiliary neoplasm, low risk/grade
PanHigh: pancreatobiliary neoplasm, high risk/grade
PCF: pancreatic cystic fluid
PCL: pancreatic cystic lesion
PDAC: pancreatic ductal adenocarcinoma
ROC: receiver operating characteristic
SCN: serous cystic neoplasm
SPN: solid pseudopapillary neoplasm
WHO: World Health Organization
