# Supplementary material for: Single GNAS Droplet-Based Digital Polymerase Chain Reaction Analysis of Pancreatic Cyst Fluid: An Effective Up-Front Strategy for Mucinous Cyst Diagnosis by Endoscopic Ultrasound-Guided Fine-Needle Aspiration
Source: Clin Transl Gastroenterol. 2025 Jul 7;16(9):e00887. doi: 10.14309/ctg.0000000000000887 (PMC12456567; doi:10.14309/ctg.0000000000000887)
Supplement: Supplementary file 1 [file ct9-16-e00887-s001.pdf]

SUPPLEMENTARY TABLES AND SUPPLEMENTARY FIGURES

Table S1. Frequency of *GNAS* mutations in iPCLs at level 1 and association with the absence or presence of one mucinous criteria

	**Total** n	***GNAS*-mutated** n (%)	***GNAS* wild-type** n (%)
All iPCL	48	16 (33)	32 (67)
No mucinous criteria	26	6 (23)	20 (77)
One mucinous criteria	22	10 (45)	12 (55)
Non ductal communication	44	14 (32)	30 (68)
Ductal communication	4	2 (50)	2 (50)
Unifocality	38	13 (34)	25 (66)
Multifocality	10	3 (30)	7 (70)
Non viscous CF	40	11 28)	29 (73)
Viscous CF	7	5 (71)	2 (29)
PCF CEA < 192 ng/mL	38	13 (34)	25 (66)
PCF CEA > 192 ng/mL	1	0 (0)	1 (100)
Non-mucinous cytology	36	13 (36)	23 (64)
Mucinous cytology	0	0 (0)	0 (0)

PCF: Pancreatic Cyst Fluid

Table S2. Pathological diagnosis of surgically resected cases

**Surgically resected PCLs**	***GNAS*-mutated n (%)**	***GNAS* wild-type n (%)**
IPMN (n=13)	11/13 (85)	1/13 (15)
IPMN with LGD or no dysplasia (n=8)	7/8 (87)	1/8 (13)
IPMN with HGD (n=4)	3/4 (75)	1/4 (25)
IPMN with adenocarcinoma (n=1)	1/1 (100)	0
MCN (n=7)	2/7 (29)	5/7 (71)
MCN with HGD (n=1)	1/1 (100)	0
SCNs (n=1)	0	1/1 (100)
Pseudocysts (n=2)	0	2/2 (100)
pNET n=1)	0	1/1 (100)
SPN (n=1)	0	1/1 (100)

Table S3. Subclassification of non-mPCN lesions according to clinical relevance

**non-mPCN subtype**	**All n (%)**	***GNAS*-mutated n (%)**
**Benign / Non-neoplastic lesions**		
Pseudocysts	14 (10.0%)	0 (0%)
Serous cystic neoplasms (SCN)	5 (3.6%)	0 (0%)
Groove pancreatitis–associated cysts	2 (1.4%)	0 (0%)
Chronic pancreatitis–associated cyst	1 (0.7%)	0 (0%)
**Non-mucinous neoplastic lesions**		
Pancreatic ductal adenocarcinoma (PDAC)	3 (2.1%)	0 (0%)
Solid pseudopapillary neoplasm (SPN)	1 (0.7%)	0 (0%)
Pancreatic neuroendocrine tumor (pNET)	1 (0.7%)	0 (0%)
**Total non-mPCN lesions**	27 (19.3%)	0 (0%)

Table S4. Patient and cyst characteristics of IPMN cases from level 3 and association with *GNAS* mutation status

Characteristics	All (n=82)	*GNAS*-mutated (n=57, 69%)	*GNAS* wild-type (n=25, 31%)	*P*
Age at EUS-FNA, yrs (mean +/- SD)	64.7 (11.4)	66.1 (10.7)	61.6 (12.6)	0.102
Gender, male/female, n (%)	46/36 (56/44)	36/21 (63/37)	10/15 (40/60)	0.052
Incidental finding, n (%)	64 (78)	44 (77)	20 (80)	0.777
Symptoms, n (%)	18 (22)	13 (23)	5 (20)	0.777
**Acute pancreatitis,** n (%)				0.568
Yes	9 (11)	7 (12)	2 (8)	
No	73 (89)	50 (88)	23 (92)	
**Chronic pancreatitis,** n (%)				0.803
Yes	11 (13)	8 (14)	3 (12)	
No	71 (87)	49 (86)	22 (88)	
**Diabetes mellitus,** n (%)				0.540
Yes	20 (24)	15 (26)	5 (20)	
No	62 (76)	42 (74)	20 (80)	
**Non pancreatic malignancies,** n (%)	31 (38)	20 (36)	11 (44)	0.479
**Age first neoplasia,** yrs (mean +/- SD)	55.7 (10.4)	61.9 (10.4)	54.9 (11.9)	0.956
**Morphological type of IPMN**				0.386
BD-IPMN	66 (80)	45 (79)	21 (84)	
Mixed-IPMN	16 (20)	12 (21)	4 (26)	
**Size on EUS,** mm, median (IQR)	23 (15-30)	23 (16-30)	20 (13-25)	0.607
**Septations on USE,** n (%)				0.924
Present	35 (43)	24 (43)	11 (44)	
Absent	46 (57)	32 (57)	14 (56)	
**Cyst location,** n (%)				0.185
Head or uncinate	47 (57)	36 (63)	11 (44)	
Neck	8 (10)	5 (9)	3 (12)	
Body	18 (22)	9 (16)	9 (36)	
Tail	9 (11)	7 (12)	2 (8)	
**Cyst multifocality,** n (%)				0.065
Available, n	82	57	25	
Yes, n (%)	50 (61)	31 (54)	19 (76)	
No, n (%)	32 (39)	26 (46)	6 (24)	
**Cyst ductal communication,** n (%)				0.099
Available, n	82	57	25	
Yes, n (%)	55 (67)	35 (61)	20 (80)	
No, n (%)	27 (33)	22 (39)	5 (20)	
**PCF consistency,** n (%)				**0.011***
Available, n	76	54	22	
Viscous	38 (50)	32 (59)	6 (27)	
Non-viscous	38 (50)	22 (41)	16 (73)	

**PCF CEA,** n (%)				**0.003***
Available, n	49	35	14	
Mucinous (> 192 ng/mL)	22 (45)	11 (31)	11 (79)	
Non-mucinous (< 192 ng/mL)	27 (55)	24 (69)	3 (21)	
**CEA concentration** (ng/mL), median (IQR)	157 (0-39530)	94 (0.1-39530)	1441 (0-21185)	**0.0022***
**Cytology,** n (%)				0.717
Available, n	66	47	19	
Mucinous (WHO IV and V)	27 (40)	20 (42)	7 (37)	
Non mucinous (WHO I, II, III, VI)	49 (60)	27 (58)	12 (63)	
**Mucin on EUS,** n (%)				0.743
Present	22 (34)	15 (33)	7 (37)	
**IPMN with advanced neoplasia,** n (%)	9 (11)	5 (9)	4 (16)	0.335
High grade dysplasia	4 (5)	2 (3)	2 (8)	
Associated invasive carcinoma	5 (6)	3 (6)	2 (8)	

The WHO numbers (I-VI) displayed on the cytology variable indicate the diagnostic category in accordance with the revised WHO System for Reporting Pancreatobiliary Cytopathology (reference 29).

Table S5. Association analysis between MAF *GNAS* values and patient and cyst characteristics in *GNAS*-mutated IPMNs

Characteristics	Spearman's r	MAF	*P*
**Male / female,** median (IQR)		2.16 (0.26 -15.8)/1.31(0.57-5.28)	0.45
**Age**	0.1640		0.23
**Cyst size**	0.1054		0.44
**IPMN type,** BD / mixed		2.19 (0.58-13.6) / 1.22 (0.78-2.4)	0.34
**Cyst focality, unifocal / multifocal,** median (IQR)		1.99 (0.43-5.97) / 1.48(I.45-16.45)	0.95
**Cyst septations, absent / present,** median (IQR)		1.43 (0.43-5.24) / 3.56(0.55-12.38)	0.31
**Cyst fluid viscosity, non-viscous / viscous**, median (IQR)		1.44 (0.28-7.72) / 2.19(0.95-9.97)	0.30
**Cyst fluid CEA concentration, >192 /< 192 ng/ml,** median (IQR)		(0.75(0.16-4.99) / 2.28(0.95-9.97)	0.26
**mPCL / iPCL at level 1,** median (IQR)		1.74 (0.32-11.16) / 2.4 (0.58-6.91)	0.55
**Advanced neoplasia, present / non present**, median (IQR)		2.26 (0.26-6.66) / 2.13 (0.68-12.38)	0.85

IQR: interquartile range; MAF: mutant allele frequency

Table S6. Predictive value of mucinous criteria and *GNAS* mutations to discriminate IPMN in the whole cohort of PCLs (n=140)

	***GNAS*-mutated**	**Cyst multifocality**	**Cyst ductal communication**	**Viscous PCF**	**[PCF CEA] >192 ng/mL**	**[PCF CEA] >41/ng/mL**	**Mucinous cytology**
**Sensitivity, %**	70	61	67	50	45	71	40
**Specificity, %**	100	78	92	96	97	84	98
**PPV, %**	100	82	93	95	96	85	96
**NPV, %**	67	56	64	54	59	70	49
**AUC**	0.85	0.70	0.80	0.73	0.71	0.78	0.69

PPV: positive predictive value; NPV: negative predictive value; AUC: area under the curve

Table S7. Predictive value of mucinous criteria and *GNAS* mutations to discriminate mPCNs -IPMNs and MCNs- in the subcohort of PCLs with histopathological confirmation (n=25)

		GNAS-mutated	Cyst multifocality	Ductal cyst communication	Viscous PCF	[PCF CEA] >192 ng/mL	[PCF CEA] >42 ng/mL	Mucinous cytology
**mPCL vs non-mPCL**	**Sensitivity, %**	65	15	29	68	20	67	47
	**Specificity, %**	100	83	100	100	100	75	100
**IPMN vs non-IPMN**	**Sensitivity, %**	85	23	62	92	0	50	44
	**Specificity, %**	85	92	100	71	70	40	86
**MCN vs non-MCN**	**Sensitivity, %**	29	0	0	29	50	83	29
	**Specificity, %**	39	78	56	35	100	60	62

Table S8. Sensitivity of GNAS mutations for diagnosing IPMN and mPCNs

	**Total cases**	***GNAS-mutated cases***	**Sensitivity (%)**
All IPMN	82	57	70
Resected IPMN	13	11	85
All mPCNs	89	59	66
Resected mPCNs	20	13	65

Table S9. Concordance between *GNAS* mutation results and cytology in 113 patients with available cytologic evaluation

	***GNAS* mutant**	***GNAS* wild-type**	**Total**
Mucinous cytology	20	9	29
Non-mucinous cytology	29	55	84
Total	49	64	113

Overall concordance: 66.4% (n=75)

Agreement expected by chance: 53.2% (n=60.2)

Cohen's Kappa coefficient: 0.28 (fair)

Table S10. Concordance between *GNAS* mutation results and cytology in 21 resected patients with available cytologic evaluation

	***GNAS* mutant**	***GNAS* wild-type**	**Total**
Mucinous cytology	4	2	6
Non-mucinous cytology	3	12	15
Total	7	14	21

Overall concordance: 76.2% (n=16)

Agreement expected by chance: 57.1% (n=12)

Cohen's Kappa coefficient: 0.44 (moderate)

Table S11. Concordance between *GNAS* mutation results and CEA levels (>192 ng/mL) in 94 patients with available CEA data

	***GNAS* mutant**	***GNAS* wild-type**	**Total**
CEA >192 ng/mL	12	14	26
CEA ≤192 ng/mL	25	43	68
Total	37	57	94

Overall concordance: 58.5% (n=55)

Agreement expected by chance: 54,7% (n=51.5)

Cohen's Kappa coefficient: 0.08 (slight)

Table S12. Concordance between *GNAS* mutation results and CEA levels (>192 ng/mL) in 17 resected patients with available CEA data

**Columna1**	***GNAS* mutant**	***GNAS* wild-type**	**Total**
CEA >192 ng/mL	1	2	3
CEA ≤192 ng/mL	6	8	14
Total	7	10	17

Overall concordance: 52.9% (n=9)

Agreement expected by chance: 55.7% (n=9.5)

Cohen's Kappa coefficient: 0.06 (slight)

Table S13. Concordance between *GNAS* mutation results and viscous fluid in 130 patients with available macroscopic description of cys fluid

Columna1	*GNAS* mutant	*GNAS* wild-type	Total
Viscous	32	10	42
Non-viscous	24	64	88
Total	56	74	130

Overall concordance: 73.8% (n=99)

Agreement expected by chance: 52.4% (n=68.2)

Cohen's Kappa coefficient: 0.45 (moderate)

Table S14. Concordance between *GNAS* mutation results and viscous fluid in resected patients with available macroscopic description of cys fluid

Columna1	*GNAS* mutant	*GNAS* wild-type	Total
Viscous	10	3	13
Non-viscous	2	8	10
Total	12	11	23

Overall concordance: 78.3% (n=18)

Agreement expected by chance: 50.3% (n=11.6)

Cohen's Kappa coefficient: 0.56 (moderate)

Table S15. Clinical outcomes of mPCN according to *GNAS* mutation status

**Variable**	***GNAS*-mutated (n=59)**	***GNAS* wild-type (n=30)**	***P***
**AN† at diagnosis**	5 (8.5%)	4 (13.3%)	-
Resected at diagnosis	4/5 (80%)	2/4 (50%)	-
Additional pancreatic surgeries (with no AN)	7	5	-
**Patients under surveillance**	**47**	**21**	-
AN detected during follow-up ‡	1 (2.1%)	2 (9.5%)	0.22
Median follow-up from EUS-FNA, years (IQR)	5.5 (4-7)	5.0 (3-7)	-
**Total AN (diagnosis + follow-up)**	6 (10.2%)	6 (20.0%)	0.21

† Advanced neoplasia (AN) was defined as the presence of high-grade dysplasia or invasive carcinoma confirmed by cytology or surgical pathology, or clinical/imaging evidence of malignancy (e.g., locally advanced or metastatic disease).
“At the time of EUS-FNA” refers to diagnosis based on initial evaluation. “During follow-up” includes new AN events detected after EUS-FNA. Median follow-up is reported from the date of EUS-FNA.
‡ Among the three patients who developed pancreatic cancer during follow-up: one GNAS-mutated patient developed a locally advanced tumor after 28 months of surveillance; two GNAS wild-type patients developed invasive carcinoma after 12 and 36 months, respectively.

Supplementary Figures

Figure S1. Prevalence and mutant allele frequency (MAF) of *GNAS* mutations in pancreatic cyst fluid samples (PCF) of 82 IPMNs recognized at diagnostic level 3. **(A)** Percentage of *GNAS*-mutated PCF samples in IPMN. **(B)** Percentage of *R201C* and/or *R201H GNAS* point mutations identified by ddPCR in 57 mutated IPMN cases. **C.** Violin plots showing MAF of *GNAS* point mutations *R201C* and/or *R201H*. The *R201C+H* category represents MAF values for individual mutations when present as single events and the sum of MAF values of both mutations when they occur concurrently. **D**. Bar graph depicting the cumulative frequency of *GNAS* MAF in mutated cases.

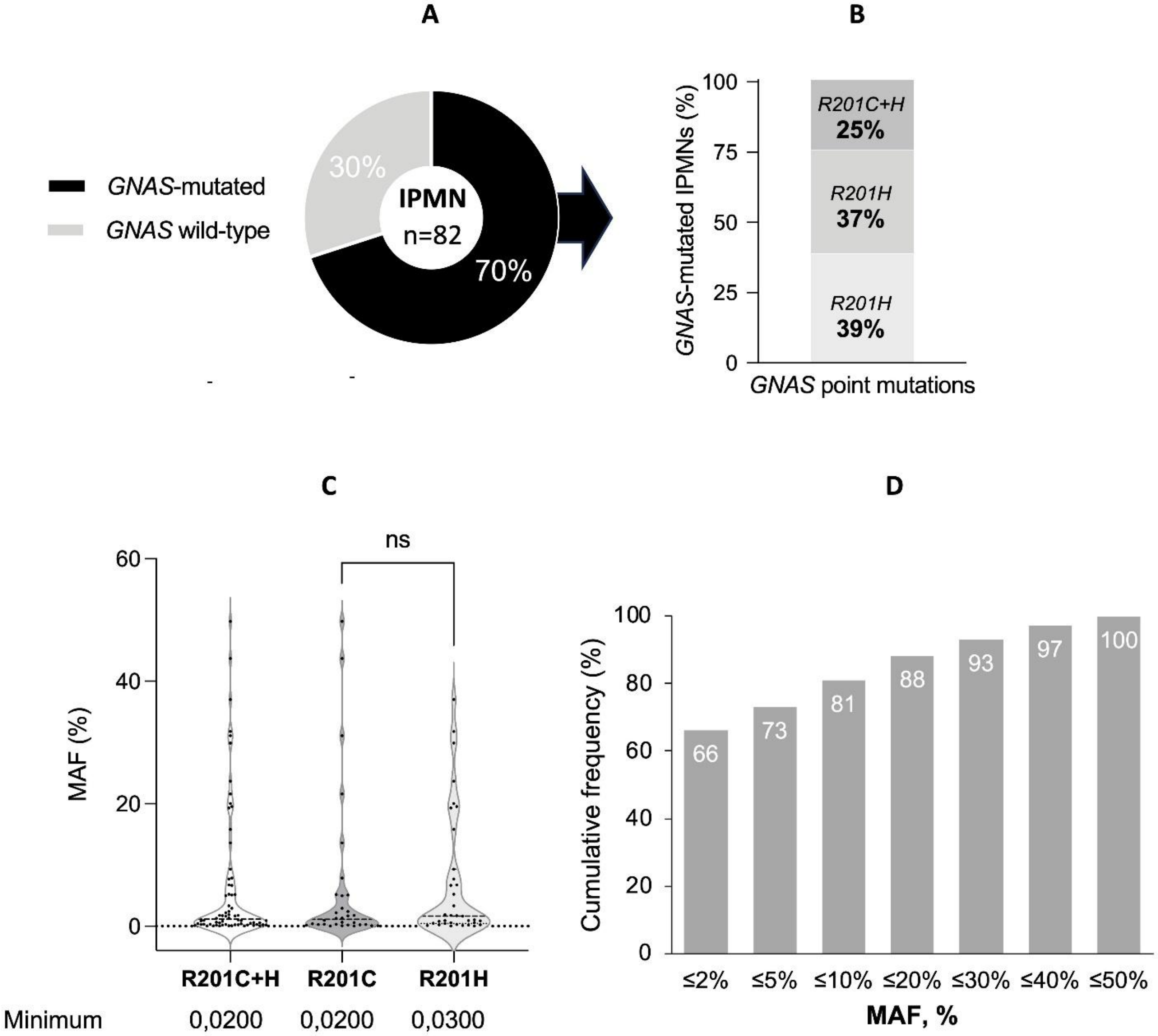

	R201C+H	R201C	R201H
Minimum	0,0200	0,0200	0,0300
25% Percentile	0,260	0,253	0,420
**Median**	**1,15**	**1,11**	**1,64**
75% Percentile	6,71	5,07	9,32
Maximum	49,8	49,8	37,0

Figure S2. Comparison of pancreatic cyst fluid (PCF) CEA concentrations between *GNAS*-mutated and wild-type IPMNs. Median CEA levels were significantly lower in *GNAS*-mutated cases (94 ng/mL vs. 1441 ng/mL; $P = 0.0022$). Data are presented as a box-and-whisker plot showing medians and interquartile ranges on a logarithmic scale.

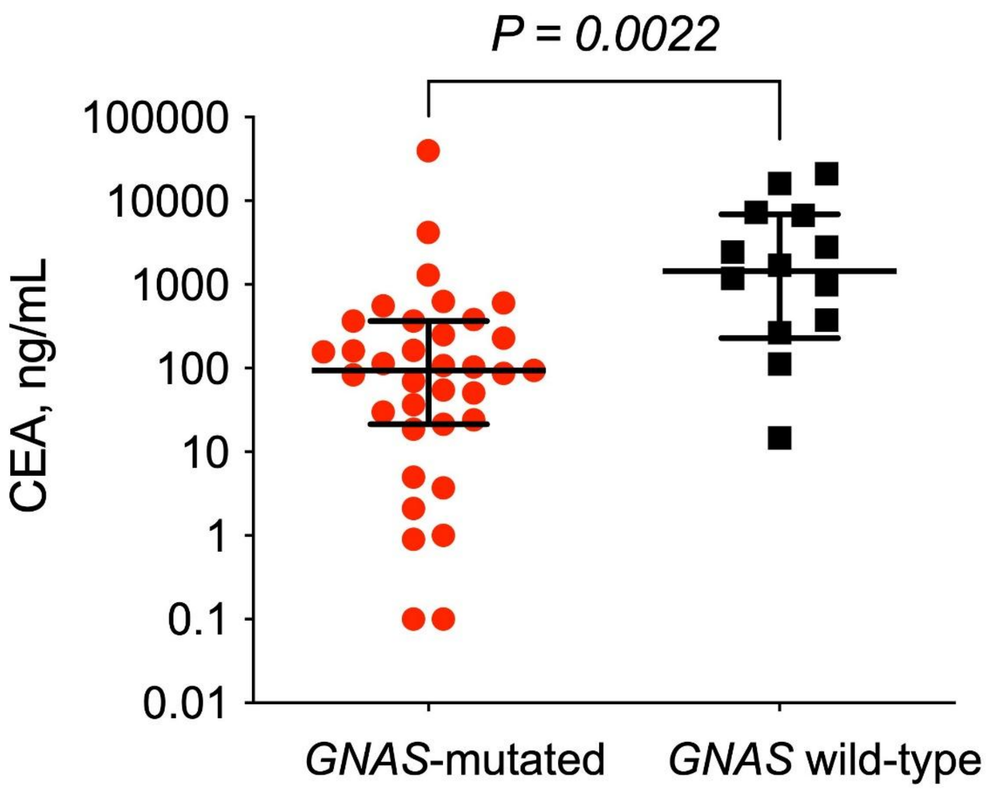

	GNAS-mutated	*GNAS* wild-type
Number of values	35	14
Minimum	0.1000	0.000
25% Percentile	21.30	228.0
Median	94.00	1441
75% Percentile	366.0	6874
Maximum	39530	21185
